# Modified (−)-gallocatechin gallate-enriched green tea extract rescues age-related cognitive deficits by restoring hippocampal synaptic plasticity

**DOI:** 10.1016/j.bbrep.2022.101201

**Published:** 2022-01-17

**Authors:** Ji-Woong Ahn, Sohyun Kim, Sukjin Ko, Young-Hwan Kim, Ji-Hyun Jeong, Seungsoo Chung

**Affiliations:** a BnH Research Co., LTD., Goyang-si, Gyeonggi-do, 10594, Republic of Korea; b Brain Korea 21 Plus Project for Medical Science, Department of Physiology, Yonsei University College of Medicine, Seoul, 03722, Republic of Korea

**Keywords:** −)-Gallocatechin gallate, Aging, Hippocampus, Long-term potentiation, Memory, aCSF, artificial cerebrospinal fluid, DIC, differential interference contrast, DMSO, dimethyl sulfoxide, EGCG, (−)-epigallocatechin-3-gallate, fEPSPs, field excitatory postsynaptic potentials, GCG, (−)-gallocatechin gallate, GTE, conventional green tea extract, HPLC-PDA, high performance liquid chromatography photometric diode array, HTP-GTE, GCG-enriched green tea extract, LTP, long-term potentiation, NMDARs, N-Methyl-d-aspartate receptors, MTT, methylthiazolyldiphenyl-tetrazolium bromide, MWM, Morris water maze, PKA, protein kinase A, SC, Schaffer collateral, TTX, tetrodotoxin

## Abstract

*Aging* leads to cognitive impairments characterized by reduced hippocampal functions that are associated with impairment of long-term potentiation of CA1 synapses. *Here, we assessed the safety and efficacy of* modified (−)-gallocatechin gallate (GCG)-enriched green tea extract (HTP-GTE) *in ameliorating the cognitive dysfunctions in late middle-aged murine model.* We developed a novel HTP-GTE that was enriched with GCG via epimerization that involved heating. We compared the effects of oral administrations of conventional green tea and HTP-GTE in young and aged male C57/BL6 mice, and examined the changes in the hippocampal functions related to aging process. The functional outcome was assessed by the electrophysiological experiments to measure the long-term potentiation (LTP). HTP-GTE improved the age-related cognitive impairments via restoring long-term synaptic plasticity. We also identified that GCG was the main active component responsible for the HTP-GTE effect. The main molecular pathway in ameliorating the age-related cognitive dysfunctions involved protein kinase A (PKA) which was shown to be modulated by HTP-GTE. Thus, HTP-GTE has a therapeutic potential as a dietary supplement which may aid to rescue the impaired cognitive functions at the early phase of aging process through the modulation of LTP threshold.

## Highlights

● HTP-GTE has a potential to improve the age-related impairment of hippocampal-dependent spatial memory function.
● The active ingredient in the HTP-GTE is GCG.
● Cognitive dysfunction at the beginning of aging process can be rescued by GCG-enriched HTP-GTE.

## 1 Introduction

Physiological brain aging is often accompanied by reduced cognitive function that directly reflects the deteriorated changes in long-term potentiation (LTP) of synaptic strength in the hippocampus [1,2]. LTP of the Schaffer collateral (SC)-CA1 synapse relies on Ca^2+^ through N-Methyl-d-aspartate receptors (NMDARs) and decreases with aging [3]. The aging-dependent decrease in the expressions and functions of protein kinase A (PKA) in CA1 inhibit NMDAR-dependent LTP at hippocampal SC-CA1 synapse [3,4]. Aging is, thus, closely associated with the reductions in the synaptic plasticity [1,2,5].

Green tea (*Camellia sinensis*) ameliorates cognitive impairment by normal aging or neurodegeneration [6,7]. (−)-epigallocatechin-3-gallate (EGCG) is one of major catechin components in green tea [8]. However, the green tea-induced effects on cognitive function have been inconsistent in different reports because the concentration and constituents of green tea vary between the studies depending on the manufacturing process. There is a low content of EGCG in green tea due to high conversion rate of EGCG to its epimer, (−)-gallocatechin gallate (GCG), during the heated brewing processes [9]. Furthermore, GCG is reported to be more structurally stable than EGCG [9] while EGCG is known to cause hepatotoxicity [10].

We developed a novel and safe GCG-enriched green tea extract called HTP-GTE. GCG, an epimer of EGCG, was enriched by heated epimerization [8,11]. The regulatory effects of HTP-GTE on synaptic plasticity of late middle-aged 16-month-old mice was tested. We examined the effect of individual catechin components in the induction of hippocampal LTP at SC-CA1.

## 2 Materials and methods

### 2.1 Animals

C57/BL6 mice were housed under standard housing conditions with 5–6 mice per cage. The mice had free access to food and water and were housed under a 12-h light/dark cycle at temperature controlled around 23 °C. Each experiment was carried out with 5–7 mice per animal group. 10-week-old mice were assigned as ‘young’ adult male group while the 16-month-old mice were allocated as the ‘late middle-aged’ male group. Animal care was performed in accordance with the Yonsei University College of Medicine Animal Care (Project license number: #2017–0070) and Use Committee or the NIH Guide for the Care and Use of Laboratory Animals.

### 2.2 Preparation of high temperature processed-green tea extracts (HTP-GTE)

Fresh green tea (*Camellia sinensis*, CS) leaves were collected in spring from Osulloc Tea Garden in Jeju, Korea. CS leaves were dried and extracted twice with 50% aqueous ethanol at 60 °C for 3 h. The decaffeination process of 50% aqueous ethanol extract was carried out with activated carbon and incubated at 1.2 atm under aqueous conditions for 5 h to gain the high temperature processed-green tea extracts (HTP-GTE). After the heating process, HTP-GTE was concentrated with a rotary evaporator (Buchi R200, Flawil, Switzerland) *in vacuo* and stored at −20 °C prior to HPLC analysis.

### 2.3 Preparation of GCG and GCG-free HTP-GTE

(−)-gallocatechin gallate (GCG) purchased from Wako pure chemical industries (Tokyo, Japan) and GCG-free HTP-GTE was provided by the AmorePacific CO R&D center (Yongin, South Korea). Isolation of GCG was done by using β-cyclodextrin-bonded silica column, and the equivalent amount of EGCG in the GTE was achieved with the addition of pure EGCG.

### 2.4 Analysis of catechins by HPLC-PDA analysis

HTP-GTE was analyzed by HPLC-PDA (Alliance 2695 system, Waters, USA) using a ThermoSyncronis C18 column (250 × 4.6 mm, I.D., 5 μm; Thermo Fisher Scientific Inc.). The mobile phases were 0.1% acetic acid in water for solvent A and acetonitrile for solvent B. The gradient elution was 90% A + 10% B at 0–10 min, 85% A + 15% B at 10–30 min, 80% A + 20% B at 30–53 min, 5% A + 95% B at 53–55 min, 90% A + 10% B at 55–60 min with a flow rate of 1.0 mL/min. The sample volume for injection was 10 μl, and the measurement was carried at the UV wavelength of 280 nm.

### 2.5 Preparation of hippocampal slice

The hippocampal slices (400 μm thickness) from young adults (10-week-old) and late middle-aged (16-month-old) mice were prepared after they were orally administered with or without green tea extracts (i.e. vehicles) for 30 days. Under isoflurane (5% isoflurane, 95% O_2_) anesthesia, mice were transcardially perfused with artificial cerebrospinal fluid (aCSF) with the following compositions: (in mM): 195.5 sucrose, 2.5 KCl, 1 NaH_2_PO_4_, 32.5 NaHCO_3_, 11 glucose, 2 Na pyruvate, and 1 Na ascorbate (Sigma-Aldrich, St. Louis, MO, USA) bubbled with 95% O_2_/5% CO_2_ at pH of 7.4. Then, the brain was extracted and the sample slices were immediately cut on a vibratome (Leica biosystems, Wetzlar, Germany). The slices were transferred to the pre-incubation solution with the following concentrations in mM: 119 NaCl, 2.5 KCl, 1 NaH_2_PO_4_, 26.2 NaHCO_3_, 11 glucose, 2 Na pyruvate, 1 Na ascorbate, 3 MgSO_4_, and 1.5 CaCl_2_ at 35 °C for 15 min. The slices were transferred to an incubation chamber with aCSF at room temperature for 1 h.

### 2.6 Electrophysiology

Details of the methods were mentioned in the articles by Franks et al. and Yu et al. [12,13]. Briefly speaking, the field recordings were carried out at the stratum radiatum of the CA1 region using an extracellular glass pipette (3–5 MΩ) that was filled with aCSF. A bipolar electrode (FHC, Bowdoin, ME, USA) was positioned on the Schaffer collateral-CA1 (SC) for stimulation. At 4 × magnification, The SC circuit was localized under the differential interference contrast (DIC) microscopy. The circuit was verified by the evoked field excitatory postsynaptic potentials (fEPSPs) at CA1 synapses via SC input stimulation. Before the start of all the experiments (30–300 μA), the test stimulation in all fEPSP experiments was done and a test-pulse stimulation strength that evoked 50% of the maximum fEPSP was used. After the 30-min baseline recording of synaptic responses, the long-term potentiation (LTP) was induced by high-frequency stimulation (HFS; 100 Hz, 4 trains, 1 s duration, 20 s inter-train interval). The results were recorded every 10 s for 1 h using the Axopatch 1D amplifier (Molecular Devices, Sunnyvale, CA, USA). The recordings were digitized at 10 KHz, and filtered at 2 KHz with Digidata 1322A and pClamp 10.0 software (Molecular devise, Sunnyvale, CA, USA). The pipette solution of the electrode was prepared as follows: (concentration in mM: 135 Cs methane sulfonate, 8 NaCl, 10 HEPES, 0.5 EGTA, 4 Mg-ATP, 0.3 Na-GTP, and 5 QX-315 Cl; pH 7.25 with CsOH, 285 mOsm) to measure excitatory postsynaptic currents (EPSCs), and the currents were recorded under the condition with 1 μM tetrodotoxin (TTX, Tocris, Bristol, England) to block the action potentials propagated by sodium currents.

### 2.7 Western blotting

The mice were anesthetized under isoflurane (5% isoflurane, 95% O2) and perfused with artificial cerebrospinal fluid (aCSF) solution. The hippocampus was isolated and homogenized for 10 min with lysis buffer (1% Triton X-100, 0.32 M sucrose in HEPES solution) with Halt protease/phosphatase inhibitor cocktail (Thermo Fisher Scientific, Waltham, CA, USA). Protein concentration was measured by BCA Protein Assay Kit (Thermo Fisher Scientific, Waltham, CA, USA). The same amounts of protein were loaded onto a 12% sodium dodecyl sulfate (SDS)-polyacrylamide gel and transferred onto 0.45-μm size pore polyvinylidene fluoride membranes. Blocking procedure was done with 5% non-fat dry milk plus 0.1% Tween 20 for 2 h, and immunoblotted with primary antibody of PKA (1:500, Thermo Fisher Scientific, Waltham, CA, USA) and β-actin for normalization (1:3000, SCBT, Dallas, TX, USA). All full blots are represented in the Supplementary Figs. S7 and S8.

### 2.8 Cell viability test with methylthiazolyldiphenyl-tetrazolium bromide (MTT) assay

Hippocampal neurons were cultured in the 24-well plate with the confluence of 2 × 10^6^ cells per well for 14 days at 37 °C in the incubator with mixed gas (95% O_2_ and 5% CO_2_). After the full maturation, the neurons were treated with GTE or HTP-GTE for 2 h, then, the medium was changed with fresh Neurobasal medium containing diluted methylthiazolyldiphenyl-tetrazolium bromide (MTT) (Sigma Aldrich, St. Louis, MO, USA) (10% MTT) for further 2 h at 37 °C. After the reaction, the media was removed and 200 μL dimethyl sulfoxide (DMSO) was added to dissolve the formazan crystals. MTT results were measured via the light absorbance at 570 nm using a microplate reader [14], and cell viability data was acquired by the ratio of optical density of the treated group over the control group.

### 2.9 Statistics

All data was reported as mean ± SEM. Statistical analysis was done using one-way ANOVA followed by Tukey's *post hoc* test. For MWM test, we used the repeated measures of two-way ANOVA (Please refer to the supplementary method for further details). Data were analyzed using Prism 5.0 software (GraphPad, La Jolla, CA, USA) and data with p < 0.05 was considered statistically significant.

## 3 Results

### 3.1 HTP-GTE rescued age-related synaptic impairments via recovering the depressed synaptic strength at SC-CA1 synapses in 16-month-old mice

**Fig. 1:**
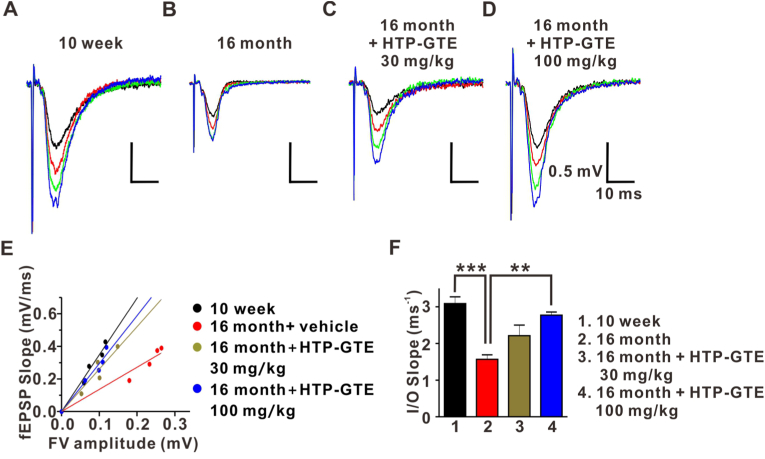
HTP-GTE augmented and rescued the impaired synaptic strength in 16-month-old mice. **A-D:** Representative hippocampal fEPSPs at four increment points of stimulus intensities. **E:** The Input and Output (I/O) relationship correlated to the recorded fEPSPs in A-D. **F:** The mean slope I/O relationship for the mice at the ages of 10 weeks, 16 months, 16 months + HTP-GTE 30 mg/kg, and 16 months + HTP-GTE 100 mg/kg group (10 week: 3.09 ± 0.18, n = 6 slices/3 mice; 16 month: 1.57 ± 0.12, n = 6 slices/3 mice; 16 month + HTP-GTE 30 mg/kg: 2.21 ± 0.29, n = 6 slices/3 mice; 16 month + HTP-GTE 100 mg/kg: 2.77 ± 0.09, n = 6 slices/3 mice). All data are represented as mean ± SEM (One-way ANOVA Tukey's post hoc test, **p < 0.01, ***p < 0.001).

The effect of HTP-GTE on the hippocampal synaptic plasticity of late middle-aged mice were investigated by, firstly, comparing the synaptic strengths recorded as fEPSPs at SC-CA1 circuits in the acute hippocampal brain slices. The synaptic strength at the SC-CA1 circuit was more significantly reduced in 16-month-old mice than in 10-week-old control. However, the synaptic strength was notably recovered in a dose-dependent manner to the level observed in the young adult mice when HTP-GTE was fed to 16-month-old mice (Fig. 1A–F). We compared the synaptic strengths in each group by measuring the slope of the I/O relationships (I/O slopes) obtained in Fig. 1E. The slope of the I/O relationship was analyzed by plotting the initial slope of evoked AMPAR fEPSPs versus fiber volley amplitude (an indicator of afferent fiber recruitment), over a range of stimulus intensities.

To investigate the effect of modified green tea and conventional green tea on the synaptic alterations in the murine hippocampi, we compared the effects of two different types of green tea on the synaptic strengths at SC-CA1 circuits in the acute hippocampal brain slices. We confirmed that there was no difference in the fEPSPs between HTP-GTE and GTE at a dose of 30 mg/kg. However, the synaptic strength was evidently stronger in the mice that received 100 mg/kg HTP-GTE than in the mice received 100 mg/kg GTE (Fig. S1).

### 3.2 HTP-GTE rescued age-related synaptic impairments by restoring LTP at SC-CA1 synapses in 16-months old mice

**Fig. 2:**
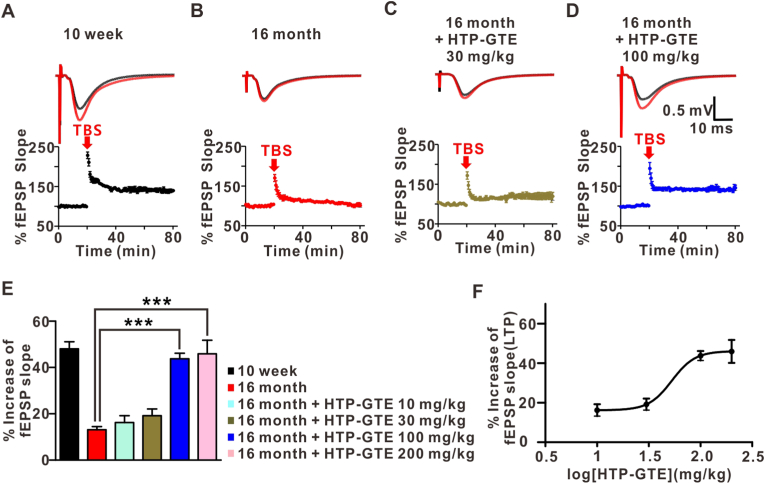
HTP-GTE restored LTP at SC-CA1 synapses in 16-month-old mice. **A-D:** Top: representative fEPSPs before (average of 20 traces, black line) and after (average of 180 traces, red line) high-frequency stimulus. Bottom: mean time courses for fEPSP amplitude during LTP induction in all groups. **E:** Quantified graph is presented (10 week: 48.00 ± 3.06, n = 6 slices/3 mice; 16 month: 13.11 ± 1.36, n = 6 slices/3 mice; 16 month + HTP-GTE 10 mg/kg: 16.21 ± 2.98, n = 6 slices/3 mice; 16 month + HTP-GTE 30 mg/kg: 19.16 ± 2.91, n = 6 slices/3 mice; 16 month + HTP-GTE 100 mg/kg: 43.74 ± 2.39, n = 6 slices/3 mice; 16 month + HTP-GTE 200 mg/kg: 45.90 ± 5.82, n = 6 slices/3 mice). All data are presented as the mean ± SEM (One-way ANOVA Tukey's post hoc test, ***p < 0.001). **F:** Dose-response relationship of % increase of fEPSP slope with HTP-GTE administration. (For interpretation of the references to colour in this figure legend, the reader is referred to the Web version of this article.)

The effect of HTP-GTE on the LTP induction was also investigated. According to the *ex vivo* results obtained from the acute hippocampal slices of 16-month-old mice fed with HTP-GTE, the reduced LTP was significantly ameliorated in 16-month-old mice (Fig. 2A–E). Unlike GTE, the LTP induction at the SC-CA1 circuit was also markedly increased in 16-month-old mice that was orally administered with 100 mg/kg HTP-GTE (Fig. S2). We found out that 100 mg/kg of HTP-GTE is the optimal dose with full effects (Fig. 2F). By orally administrating HTP-GTE, the synaptic strength were recovered almost fully in the aged mice.

### 3.3 PKA is a key regulator in the HTP-GTE-induced improvement of aged hippocampal synaptic impairments

**Fig. 3:**
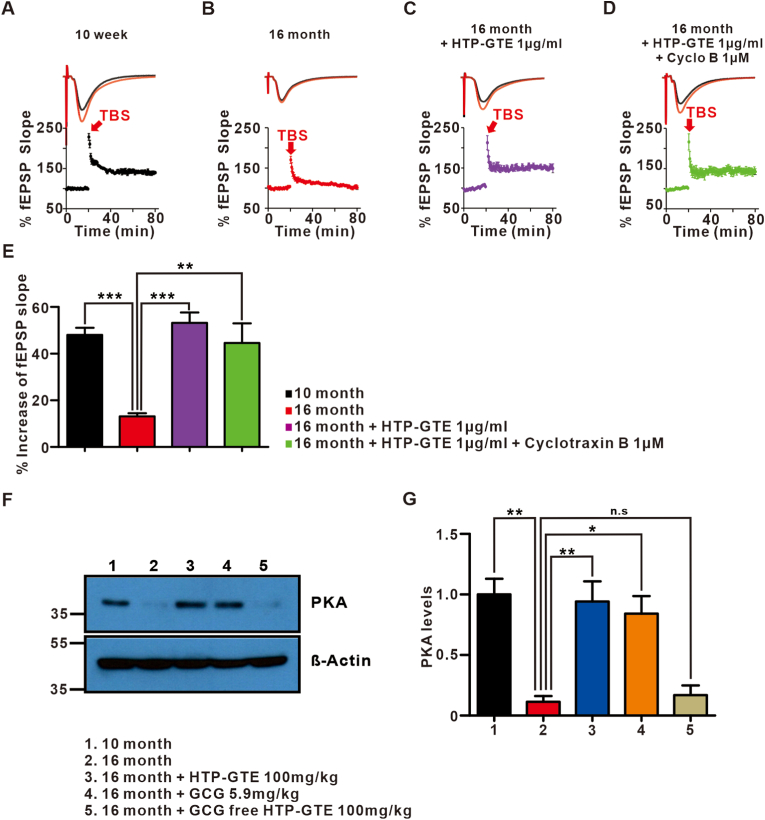
PKA, not BDNF-TrkB pathway, is an important biochemical regulator in modulating HTP-GTE-induced improvement of aged hippocampal synaptic impairments. **A-D:** Top: representative fEPSPs before (average of 20 traces, black line) and after (average of 180 traces, red line) high-frequency stimulus. Bottom: mean time courses for fEPSP amplitude during LTP induction in all groups. Recordings are presented as mean ± SEM. **E:** HTP-GTE and Cyclotraxin B (TrkB receptor antagonist) were perfused exogenously for 2 h to the brain slice samples during this *ex vivo* study. Quantified graph is presented (10 week: 48.01 ± 3.06, n = 6 slices/3 mice; 16 month: 13.11 ± 1.36, n = 6 slices/3 mice; 16 month + HTP-GTE 1 μg/ml: 53.18 ± 4.42, n = 6 slices/3 mice; 16 month + HTP-GTE 1 μg/ml + Cyclotraxin B 1 μM: 44.57 ± 8.42, n = 6 slices/3 mice). **F-G:** Western blot of PKA levels in the hippocampus from the mice at the ages of 10 weeks, 16 months, 16 months + HTP-GTE 100 mg/kg, 16 months + GCG 5.9 mg/kg, and 16 months + GCG-free HTP-GTE 100 mg/kg with the oral administration different green tea tracts daily for 4 weeks (1 ± 0.13, 0.11 ± 0.05, 0.94 ± 0.17, 0.84 ± 0.15, and 0.169 ± 0.08, respectively). Full-length blot is presented in the Supplementary Fig. S6. All data are presented as the mean ± SEM (One-way ANOVA Tukey's post hoc test, *p < 0.05, **p < 0.01, ***p < 0.001, n. s. = not significant). (For interpretation of the references to colour in this figure legend, the reader is referred to the Web version of this article.)

Our previously published report in the depression animal models, HTP-GTE modulated the BDNF-TrkB pathway which was associated with improving cognitive impairments caused by post-menopausal depression [15]. We investigated whether the same molecular mechanism could be applied in the aged models. Unlike depression-induced cognitive impairments, the age-related cognitive deficit showed different pathophysiological mechanisms and experimental results [16]. Surprisingly, the effect of improved LTP after HTP-GTE treatment was not blocked by the TrkB receptor antagonist, Cyclotraxin B (Cyclo B) (Fig. 3A–E). We deduced that a different molecular mechanism other than BDNF-TrkB pathway might be involved. Since PKA is well known to be associated with the aging-induced LTP deterioration in many literatures [[17], [18], [19], [20], [21], [22], [23]], we tested the hippocampal PKA levels in the mice with different types of green tea extracts and compared these animals to the control mice fed with vehicle. The Western blot data showed that the depressed PKA levels in the aged mice were dramatically restored after the feeding of HTP-GTE in the aged mice (Fig. 3F–G). We also discovered that GCG may be a critical player in modulating the cognitive deficits caused by aging process. This was further confirmed that there was no significant difference in the PKA levels between the vehicle-fed aged mice and the mice fed with GCG-free green tea extracts (Fig. 3G).

### 3.4 HTP-GTE is less neurotoxic than GTE with high effects

In order to compare the toxicity degrees of GTE and HTP-GTE, we investigated the neuronal cell viability of the cultured mouse hippocampal neurons via MTT assays. After the exogenous applications of HTP-GTE (Exo-HTP-GTE) or GTE (Exo-GTE) or GCG (Exo-GCG) at various doses between 0.03 and 3 μg/mL for 2 h, the viability of hippocampal neurons were assessed. The reduced levels of cell viability was observed dose-dependently. At higher doses above 1 μg/ml, the conventional GTE showed more neurotoxic effect than HTP-GTE (Fig. S3). It is noteworthy to mention that the level of cell viability was not different between the control cells with vehicle treatment and HTP-GTE-treated cells (Fig. S3). Our data strongly demonstrated that HTP-GTE enriched with GCG is less toxic than the conventional green tea.

### 3.5 (−)-Gallocatechin gallate (GCG), but not EGCG, contributes to HTP-GTE-induced improvements in synaptic impairments in aged mice

**Fig. 4:**
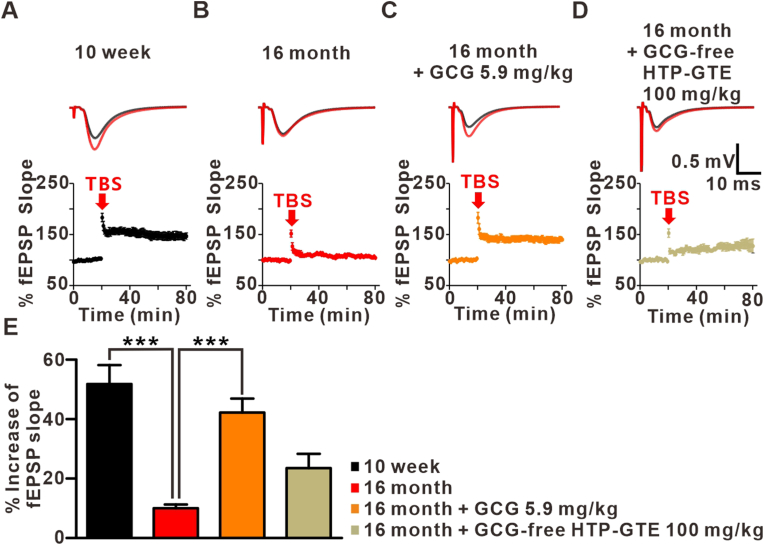
Feeding of GCG for 4 weeks restored LTP at SC-CA1 synapses in 16-month-old mice. **A-D:** Top: representative fEPSPs before (average of 20 traces, black line) and after (average of 180 traces, red line) high-frequency stimulus. Bottom: mean time courses for fEPSP amplitude during LTP induction in all groups. **E:** Quantified graph was presented (10 week: 51.84 ± 6.31, n = 6 slices/3 mice; 16 month: 10.00 ± 1.26, n = 6 slices/3 mice; 16 month + GCG 5.9 mg/kg: 42.22 ± 4.72, n = 6 slices/3 mice; 16 month + GCG-free HTP-GTE 100 mg/kg: 23.54 ± 4.80, n = 6 slices/3 mice). Data are shown as the mean ± SEM (One-way ANOVA Tukey's post hoc test, ***p < 0.001). (For interpretation of the references to colour in this figure legend, the reader is referred to the Web version of this article.)

Of many catechins in green tea, we attempted to find the key component of HTP-GTE that were dominantly responsible for improving hippocampal synaptic impairments in the aged mice. We compared the compositions of catechins in HTP-GTE and GTE by the high-performance liquid chromatography photometric diode array (HPLC-PDA). Large proportions of catechins were EGCG and EGC in both GTE and HTP-GTE. However, the epimers of EGCG and EGC, such as GCG, CG and GC, were found with higher contents in HTP-GTE than in GTE (Supplementary Table 1). Next, to investigate the effect of GCG, 16-month-old mice were orally administered with GCG and GCG-free HTP-GTE daily for 30 days, then their hippocampal LTP was measured (Fig. 4A–E). The difference in the magnitude of LTP between 10-week-old mice fed with vehicle and 16-month-old mice fed with GCG was smaller. Additionally, the LTP was reduced in the control 16-month-old mice whereas it was dramatically recovered in 16-month-old mice fed with GCG. However, 16-month-old mice fed with GCG-free HTP-GTE still showed reduced LTP (Fig. 4E). These data showed similar patterns to the LTP-related protein levels of PKA in the corresponding models (Fig. 3F–G). We also investigated the direct action of exogenous GCG treatment on the induction of LTP in the acute hippocampal slices from 16-month-old mice (Fig. S4). The acute treatment of exogenous GCG (i.e. Exo-GCG) also restored LTP in the *ex vivo* studies, whereas EGCG, CG, or GC did not.

### 3.6 Pre-synaptic transmission was not involved in the improvement of synaptic plasticity modulated by HTP-GTE or GCG

To further evaluate the changes in the pre-synaptic transmission in the animal models treated with HTP-GTE or GCG, the short-term plasticity was assessed by calculating paired-pulse ratio. We found no statistical difference between the groups treated either vehicle or green tea extracts (Fig. S5). Hence, it was demonstrated that the actions of HTP-GTE and GCG were determined by post-synaptic events.

### 3.7 GCG improved the cognitive impairment in the aged mice

The functional effects of HTP-GTE and its crucial component, GCG, on learning and memory must be investigated with *in vivo* studies. All the animals underwent Morris Water Maze test (Fig. S6). The mean escape latency was measured by analyzing the time recorded during the experiments. Unlike young 10-week-old mice, the time taken to find the platform was longer in 16-month-old mice. On Day 2, 16-month-old mice with vehicle and those fed with GCG showed significant difference in the mean escape latency. On Day 5 and 6, 16-month-old mice with vehicle showed greater differences in the mean escape latency when they were compared to the 10-week-old mice, 16-month-old mice fed with HTP-GTE, and 16-month-old mice fed with GCG only (Fig. S6A).

Moreover, the time spent in the target quadrant and the number of platform crossings by 16-month-old was less than that of young adult mice (Figs. S6B–C). However, the oral feeding of either HTP-GTE or GCG to 16-month mice dramatically recovered their abilities to perform the tasks to the level of young adults. GCG-free HTP-GTE was not effective on improving cognitive impairments. The swim velocity was measured and we found that the swim speeds were significantly slower in 16-month-old mice (Fig. S6D). However, the HTP-GTE treatment brought the performances comparable with the 10-week-old mice (Fig. S6E). In sum, these findings suggested that the rescue of impaired spatial memory function by HTP-GTE is GCG-dependent.

## 4 Discussion

As mentioned in the report by Chako et al. [24], the general green tea is well known for its beneficial effect in human health. However, the detailed mechanisms of its action to improve the diseased conditions are still under investigation by many researchers. We, hereby, reported that the normal aging process was linked with gradually impaired cognitive functions partly due to the hippocampal synaptic changes, and that this impairment could be reversed by using HTP-GTE, or its active component, GCG. Furthermore, both HTP-GTE and GCG alone were capable of restoring LTP in 16-month-old mice to the levels of LTP observed in the young adult mice. In order to find the direct mechanistic action of green tea extracts on the brain tissue, one must not rule out the blood-brain barrier (BBB) permeability of the substrates. Pervin et al. reviewed about the BBB permeability of different green tea catechins, mostly about the EGCG and its metabolites [25]. HTP-GTE and GCG were also reported to pass the BBB [17]. It is important to note that variable green tea extracts are capable of BBB permeability to reach the brain regions to potentially influence the synaptic functions.

Regarding the specific component of green tea responsible for improving cognitive function, EGCG has been initially reported to be the active polyphenol primarily responsible for them [26]. In fact, Dela Torre et al. [27], showed that EGCG treatment improved the visual memory recognition and social functioning in patients with Down syndrome. In addition, the long-term administration of green tea containing high-dose EGCG improved the cognitive functions in the rats of various ages [14,28], and delayed the memory deterioration in the late middle-aged mice [23]. However, our study demonstrated that GCG was major bioactive component in green tea that acts to modulation synaptic plasticity. The enrichement of GCG in the green tea notably showed that the depressed hippocampal synaptic transmission and plasticity was improved. The slope of the I/O relationship was analyzed by plotting the initial slope of evoked AMPAR fEPSPs versus fiber volley amplitude (an indicator of afferent fiber recruitment), over a range of stimulus intensities. The synaptic strengths were recovered in a dose-dependent manner to the level observed in the young adult mice when HTP-GTE was fed to 16-month-old mice.

The reduced LTP at SC-CA1 synapse which was seen with aging has been reported in the previous observations [3]. Aging lowers the threshold of LTP and increases the tendency for synaptic depression [1,29,30]. All of these pathophysiological process contributes to impaired memory functions in the aged individuals [17,21,31]. Therefore, it is important to explore ways to restore the depressed LTP levels. Here, the oral feeding of HTP-GTE rescued the depressed SC-CA1 LTP in the 16-month-old mice. The increase of LTP after HTP-GTE treatment was also mimicked by GCG treatment alone. Furthermore, GCG-free HTP-GTE failed to increase the LTP at SC-CA1. These findings all sum up to demonstrate that GCG is a major contributor in HTP-GTE to improve in age-related depression of hippocampal LTP. GCG-rich green tea, itself, has already been reported to have a good effect in lowering cholesterol levels in the hyperlipidemic rats [32]. GCG must have diverse effects in restoring the normal conditions in many disease models [15,32].

We demonstrated that HTP-GTE modulates the synaptic plasticity of hippocampus to increase the LTP at SC-CA1 in aged mice. Pereda et al. reported that the mean slopes of fEPSPs and paired-pulse ratios were different between the young and aged mice, and we showed similar patterns in the electrophysiological experiments [33]. According to our results, the pre-synaptic transmission was not involved in the improvement of synaptic plasticity modulated by HTP-GTE or GCG. The short-term plasticity did not ameliorate the cognitive function. The post-synaptic events affected by GCG were confirmed to be crucial in modulating the synaptic plasticity in the aging process.

Our study was conducted with late middle-aged mice. Thus, there is a limitation in this study to extrapolate the data that HTP-GTE may be effective in the groups of very old mice with severe cognitive dysfunctions, or in the animal models with progressed neurodegenerative diseases. However, at least, we can suggest that the reduced LTP at the “early” phase of aging process can be reversible, and HTP-GTE can be a powerful nutritional supplement to prevent or delay the age-related cognitive deterioration. The impaired functions of spatial memory of the mice fed with HTP-GTE were recovered to the level of young adult mice. HTP-GTE intake can be applied in humans of middle-aged populations with possibly impending cognitive deterioration by serving it as a dietary supplement to enhance the cognitive performance.

In conclusion, we demonstrated that HTP-GTE effectively and safely improved the hippocampal dysfunctions associated with aging. We also identified GCG as a crucial component that is responsible for improving the depressed cognitive function. Overall, we suggested that GCG-enriched green tea is a possible therapeutic candidate to improve age-related cognitive deficits.

## Declaration of competing interest

AmorePacific R&D Center funded this study (2017-31-0705). S. C. and J. W. are co-inventors on a patent application filed by AmorePacific CO. and Industry-Academic CO. Foundation, Yonsei University incorporating discoveries described in the manuscript. S. K declares no competing interest.

## Appendix A Supplementary data

Multimedia component 2 Multimedia component 2

figs1 figs1

figs2 figs2

figs3 figs3

figs4 figs4

figs5 figs5

figs6 figs6

figs7 figs7

figs8 figs8
