# Supplementary material for: Modified (−)-gallocatechin gallate-enriched green tea extract rescues age-related cognitive deficits by restoring hippocampal synaptic plasticity
Source: Biochem Biophys Rep. 2022 Jan 17;29:101201. doi: 10.1016/j.bbrep.2022.101201 (PMC8841891; doi:10.1016/j.bbrep.2022.101201)
Supplement: Multimedia component 1 [file mmc1.docx]

| **Compounds** | **GTE (%)** | **HTP-GTE (%)** |
| --- | --- | --- |
| Caffeine | 8.13 | 3.7 |
| Epigallocatechin (EGC) | 8.49 | 3.53 |
| Epicatechin (EC) | 2.25 | 0.95 |
| Epigallocatechin gallate (EGCG) | 15.52 | 5.86 |
| Gallocatechin gallate (GCG) | 0.19 | 5.9 |
| Epicatechin gallate (ECG) | 3.47 | 1.3 |
| Catechin gallate (CG) | 0.03 | 0.4 |
| Gallocatechin (GC) | 1.52 | 4.9 |

**Table 1. Components of GTE and HTP-GTE**.

**GTE**: conventional green tea extract; **HTP-GTE**: (-)-gallocatechin gallate (GCG)-enriched green tea extract
