# Supplementary material for: Modified (−)-gallocatechin gallate-enriched green tea extract rescues age-related cognitive deficits by restoring hippocampal synaptic plasticity
Source: Biochem Biophys Rep. 2022 Jan 17;29:101201. doi: 10.1016/j.bbrep.2022.101201 (PMC8841891; doi:10.1016/j.bbrep.2022.101201)
Supplement: Multimedia component 2 [file mmc2.docx]

**Ahn et al., BB Reports**

**Supplementary Data**

**Supplementary Materials and Methods**

1. **Morris water maze (MWM) test**

A black circular pool (150 cm diameter x 35 cm height) submerged 1 cm below the water surface was used for classical water maze trialing. The visual cues were placed on the 4 sides of the walls in the experimental room. Pool was made murky with skim milk, and temperature was maintained at room temperature around 22 ℃. Animal motion was tracked and analyzed by using EthoVision XT (Noldus Information Technology Inc.).

All the animals were trained to find a hidden platform in the pool for 6 days. The trial on the first day started by placing the mice into the pool facing the mark on the wall at one of the four quadrants. The trials finished when the mice climbed onto the platform. If the mice failed to find the platform within 1 minute, they were guided to the platform and allowed to stay on the platform for 10 seconds. This guiding trial was done only once throughout the 6-day test. From the second day onwards, the trial began by putting the mice into the pool facing the same mark that was used on the first trial day, and they were tested for their ability to find the platform within two minutes. Four trials (with a 20-minute interval) were tested every day for each mouse until the end of the MWM test. On the sixth day (last day of testing), the hidden platform was removed. The time spent in the target quadrant, the platform crossing number, and swimming velocity were visually recorded using an infrared video camera.

1. **Electrophysiological evaluation of short-term plasticity**

Pre-synaptic transmission was assessed through the calculation of paired-pulse ratio with inter-stimulus intervals of 100 ms. Ten successive responses were recorded. To ensure slice viability and stable recording conditions within and between slices, the membrane input resistance, membrane potential, and electrode resistance were strictly monitored while other experimental parameters remained unchanged.

**Supplementary figure captions**

**Fig. S1. HTP-GTE is better at rescuing the impaired synaptic strength in 16-month-old mice when compared with GTE.**

**A-D:** Representative traces of fEPSPs from hippocampal slices from representative experiments at four increasing stimulus intensities. **E-F:** The scatter plot of the Input and Output (I/O) relationship corresponding to the recorded fEPSPs in A-D. **G:** The average of slope I/O relationship for 16 month + GTE 30 mg/kg, 16 month + HTP-GTE 30 mg/kg, 16 month + GTE 100 mg/kg, and 16 month + HTP-GTE 100 mg/kg group (16 month + GTE 30 mg/kg: 2.05$\pm$0.19, n = 5 slices/3 mice; 16 month + HTP-GTE 30 mg/kg: 2.21$\pm$0.29, n = 6 slices/3 mice; 16 month + GTE 100 mg/kg: 2.10 $\pm$0.16, n = 5 slices/3 mice; 16 month + HTP-GTE 100 mg/kg: 2.77$\pm$0.09, n = 6 slices/3 mice). Data are presented as mean $\pm$SEM (One-way ANOVA Tukey’s post hoc test, **p < 0.01). **H:** Dose-response relationship of I/O slope with HTP-GTE administration.

**Fig. S2. HTP-GTE, not GTE, restored LTP at Schaffer collateral-CA1 synapses in 16-month-old mice.**

**A-D:** Top: representative traces showing LTP before (average of 20 traces, black line) and after (average of 180 traces, red line) high-frequency stimulus. Bottom: average time courses for field EPSP amplitude during LTP induction in all groups. **E:** Quantified graph is shown (16 month + GTE 30 mg/kg: 18.32$\pm$3.22, n = 5 slices/3 mice; 16 month + HTP-GTE 30 mg/kg: 19.16$\pm$2.91, n = 6 slices/3 mice; 16 month + GTE 100 mg/kg: 21.57$\pm$2.12, n = 6 slices/3 mice; 16 month + HTP-GTE100 mg/kg: 43.74$\pm$2.39, n = 6 slices/3 mice). All data are represented as mean $\pm$ SEM (One-way ANOVA Tukey’s post hoc test, ***p < 0.001).

**Fig. S3. Toxicity test of HTP-GTE, GTE, and GCG which were exogenously treated on the hippocampal neuronal cells.**

**A:** Cell viability test in the primary hippocampal neurons (Control with vehicle: 100.00$\pm$ 1.19; GTE 0.03 μg/ml: 88.81$\pm$ 1.76; GTE 0.1 μg/ml: 77.83$\pm$0.73; GTE 0.3 μg/ml: 73.43 $\pm$0.30; GTE 1 μg/ml: 61.35 $\pm$0.46; GTE 3 μg/ml: 47.66 $\pm$0.55, n = 6; HTP-GTE 0.03 μg/ml: 97.39 $\pm$1.18; HTP-GTE 0.1 μg/ml: 89.88 $\pm$2.66; HTP-GTE 0.3 μg/ml: 87.04 $\pm$0.92; HTP-GTE 1 μg/ml: 81.65$\pm$0.93; HTP-GTE 3 μg/ml: 71.05$\pm$ 1.12, n = 6; GCG 0.03 μg/ml: 97.10$\pm$2.27; GCG 0.1 μg/ml: 96.39 $\pm$ 2.32; GCG 0.3 μg/ml: 100.06 $\pm$4.58; GCG 1 μg/ml: 86.55$\pm$ 2.79; GCG 3 μg/ml: 83.84 $\pm$2.41, n = 6). All data are represented as mean $\pm$ SEM (One-way ANOVA Tukey’s *post hoc* test, *p < 0.05, **p < 0.01, ***p < 0.001).

**Fig. S4. GCG is the bioactive component of HTP-GTE to improve cognitive and synaptic impairments in 16-month old mice.**

**A-D:** Top: representative traces showing LTP before (average of 20 traces, black line) and after (average of 180 traces, red line) high-frequency stimulus. Bottom: average time courses for field EPSP amplitude during LTP induction in all groups. **E:** Quantified graph is shown (16 month + GTE 30 mg/kg: 18.32$\pm$3.22, n = 5 slices/3 mice; 16 month + HTP-GTE 30 mg/kg: 19.16$\pm$2.91, n = 6 slices/3 mice; 16 month + GTE 100 mg/kg: 21.57$\pm$2.12, n = 6 slices/3 mice; 16 month + HTP-GTE 100 mg/kg: 43.74$\pm$2.39, n = 6 slices/3 mice). All data are represented as mean $\pm$ SEM (One-way ANOVA Tukey’s post hoc test, *** p < 0.001).

**Fig. S5. HTP-GTE or GCG did not make changes in the pre-synaptic transmission through the short-term plasticity.**

**A-D:** Top: representative traces showing paired-pulse ratios recorded from fEPSP amplitudes with inter-stimulus interval of 100 ms. Unlike young adults, aged mice showed no difference between the vehicle and HTP-GTE or GCG treatments. **E:** Quantified graph is shown (10 week month + vehicle: 1.77$\pm$0.15, n = 6 slices/3 mice; 16 month + vehicle: 1.19$\pm$0.05, n = 6 slices/3 mice; 16 month + HTP-GTE 100 mg/kg: 1.18$\pm$0.07, n = 6 slices/3 mice; 16 month + GCG 5.9 mg/kg: 1.17$\pm$0.06, n = 6 slices/3 mice). All data are represented as mean $\pm$ SEM (One-way ANOVA Tukey’s post hoc test, *p < 0.01, ***p < 0.001).

**Fig. S6. Morris water maze test of the control mice with vehicle treatment and 16-months old mice with the treatments of different types of green tea extracts.**

**A:** Data is presented as the representative mean escape latency on each day and is plotted as mean ± SEM (Two-way ANOVA compared to 16-month (red) mice data. ^++^p < 0.01, ^+^, *, ^x^p < 0.05). **B:** Quantification of time spent in the target quadrant (10 week: 23.6 ± 2.56, n = 10; 16 month: 14.5 ± 1.83, n = 13; 16 month + HTP-GTE 100 mg/kg: 23.6 ± 2.18, n = 10; 16 month + GCG 5.9 mg/kg: 25.4 ± 2.87, n = 9; 16 month + GCG free HTP-GTE 100 mg/kg: 16.1 ± 2.46, n = 9). **C:** Quantification of the number of crossing the platforms (10 week: 3.6 ± 0.75, n = 10; 16 month: 0.8 ± 0.19, n = 13; 16 month + HTP-GTE 100 mg/kg: 2.6 ± 0.27, n = 10; 16 month + GCG 5.9 mg/kg: 4.3 ± 0.44, n = 9; 16 month + GCG free HTP-GTE 100 mg/kg: 1.2 ± 0.43, n = 9). **D:** Comparison of the swimming velocity (cm/s) (10 week: 36.4 ± 0.74, n = 10; 16 month: 22.3 ± 3.61, n = 13; 16 month + HTP-GTE 100 mg/kg: 24.1 ± 0.39, n = 10; 16 month + GCG 5.9 mg/kg: 19.9 ± 0.57, n = 9; 16 month + GCG-free HTP-GTE 100 mg/kg: 21.1 ± 0.55, n = 9). Data of B-D are shown as mean ± SEM . Repeated-measures of two-way ANOVA was used to analyze different groups of animals during the Morris water maze training sessions. Two-way ANOVA, Tukey’s post hoc test, *p < 0.05, ***p < 0.001). **E-I:** Representative movement tracings of the swim patterns of the mice during the Morris water test.

**Fig. S7. Full western blot images of the LTP-related protein levels of PKA in the hippocampus with the treatment of different types of green tea extracts.**

The labeling numbers of each column corresponds to the following mice group: 1 = control 10-weeks old mice with vehicle treatment; 2 = control 16-month old mice with vehicle treatment; 3 = 16 months + HTP-GTE 100 mg/kg; 4 = 16 months + GCG 5.9 mg/kg; and 16 months + GCG-free HTP-GTE 100 mg/kg. The mean of the three control values was used to normalize the different experimental conditions. **A:** Representative immunoblot for PKA at approximately 43-47 kDa. **B:** Representative immunoblot for the housekeeping gene of mice β-actin at 42 kDa.

**Fig. S8. Full western blot images of the LTP-related protein levels of PKA in the hippocampus with the treatment of different types of green tea extracts.**

**Table 1. The comparison of the components of GTE and HTP-GTE.**

**CG:** Catechingallate; **ECG:** Epicatechingallate; **EC:** Epicatechin; **EGC:** Epigallocatechin; **EGCG:** Epigallocatechin gallate; **GC:** Gallocatechin**; GCG:** Gallocatechingallate; **GTE**: conventional green tea extract; **HTP-GTE**: (-)-gallocatechin gallate (GCG)-enriched green tea extract
