# Supplementary figures and images for: Modified (−)-gallocatechin gallate-enriched green tea extract rescues age-related cognitive deficits by restoring hippocampal synaptic plasticity
Source: Biochem Biophys Rep. 2022 Jan 17;29:101201. doi: 10.1016/j.bbrep.2022.101201 (PMC8841891; doi:10.1016/j.bbrep.2022.101201)

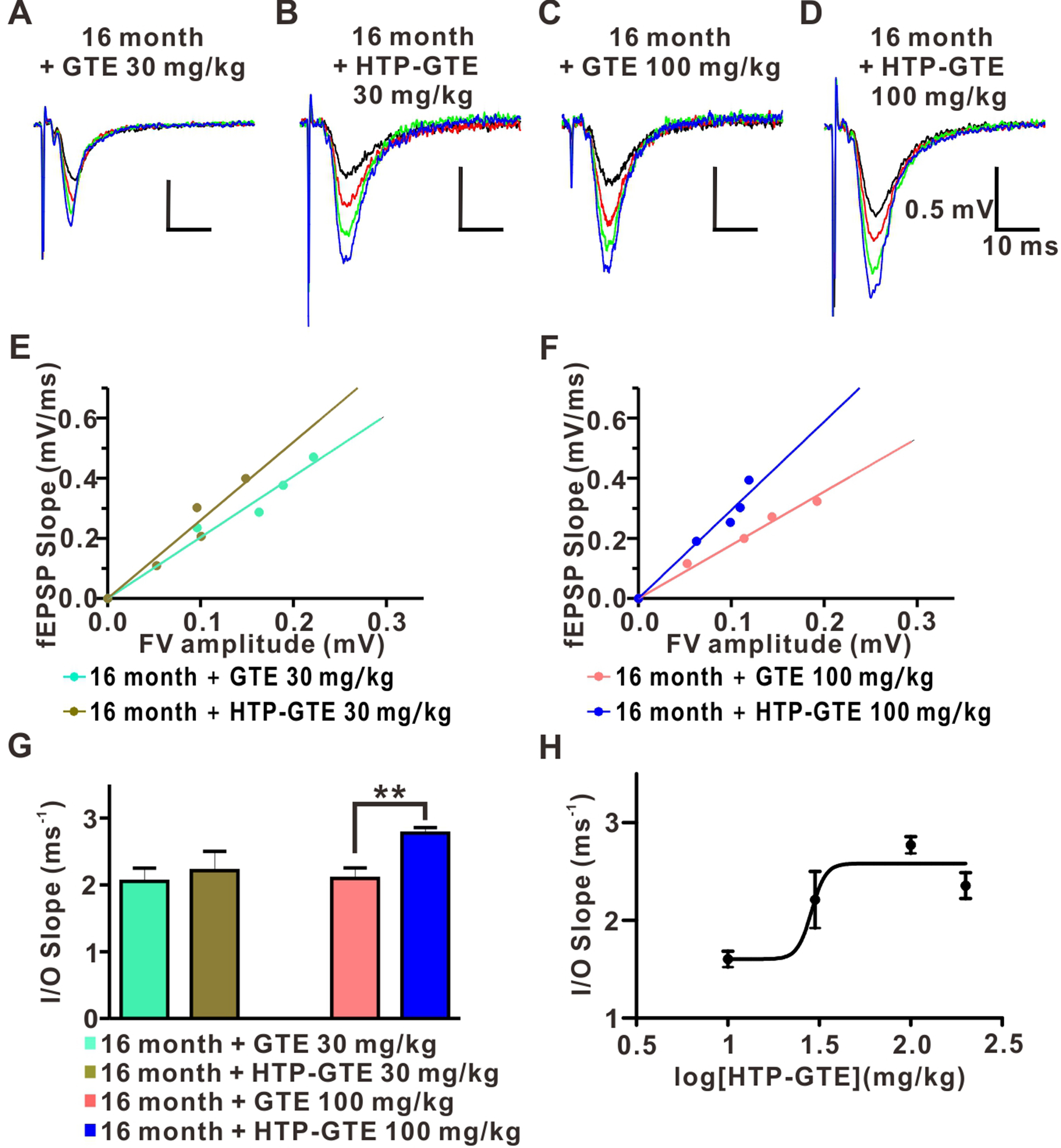

Supplement: figs1 [file mmcfigs1.jpg]

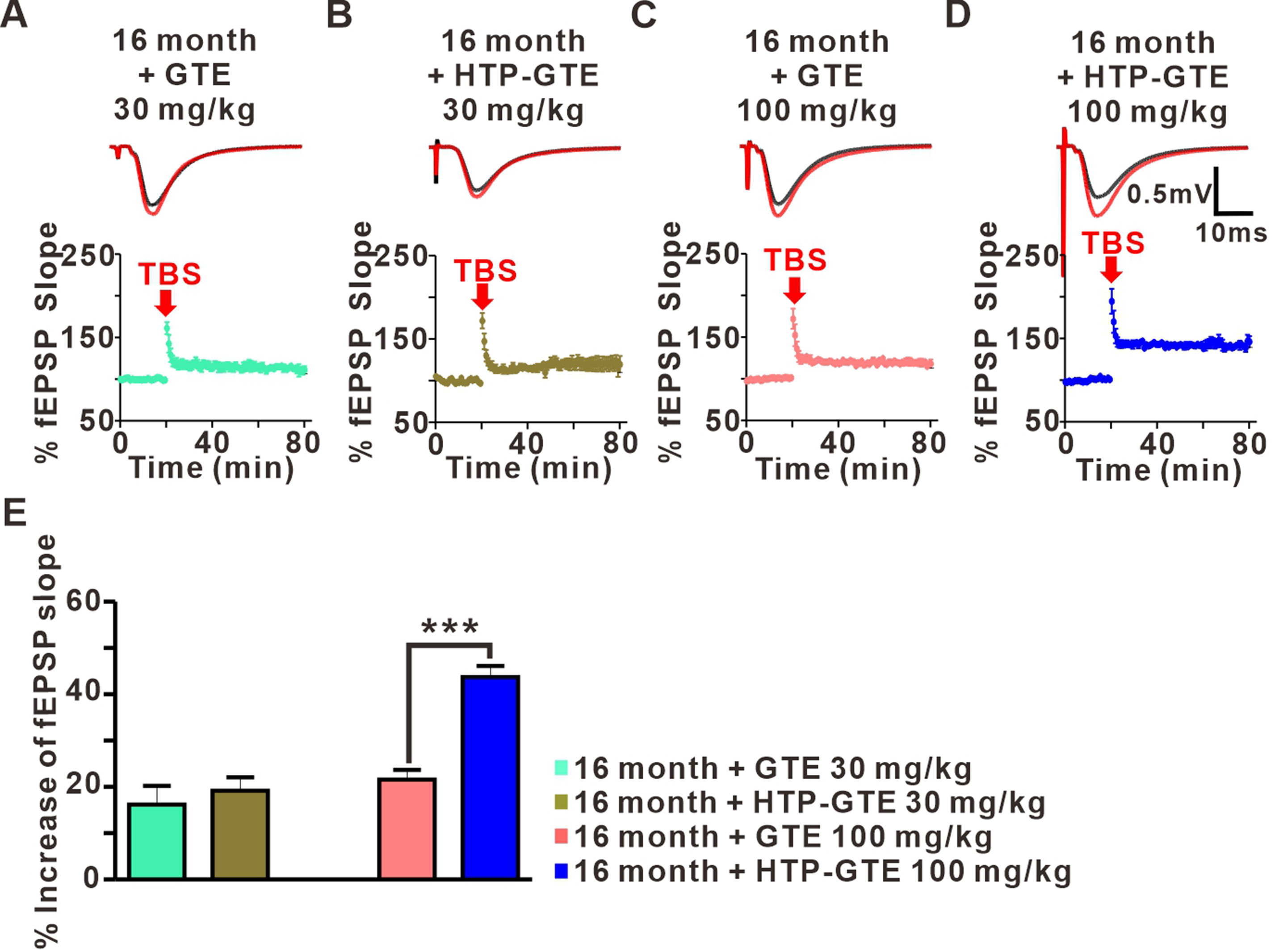

Supplement: figs2 [file mmcfigs2.jpg]

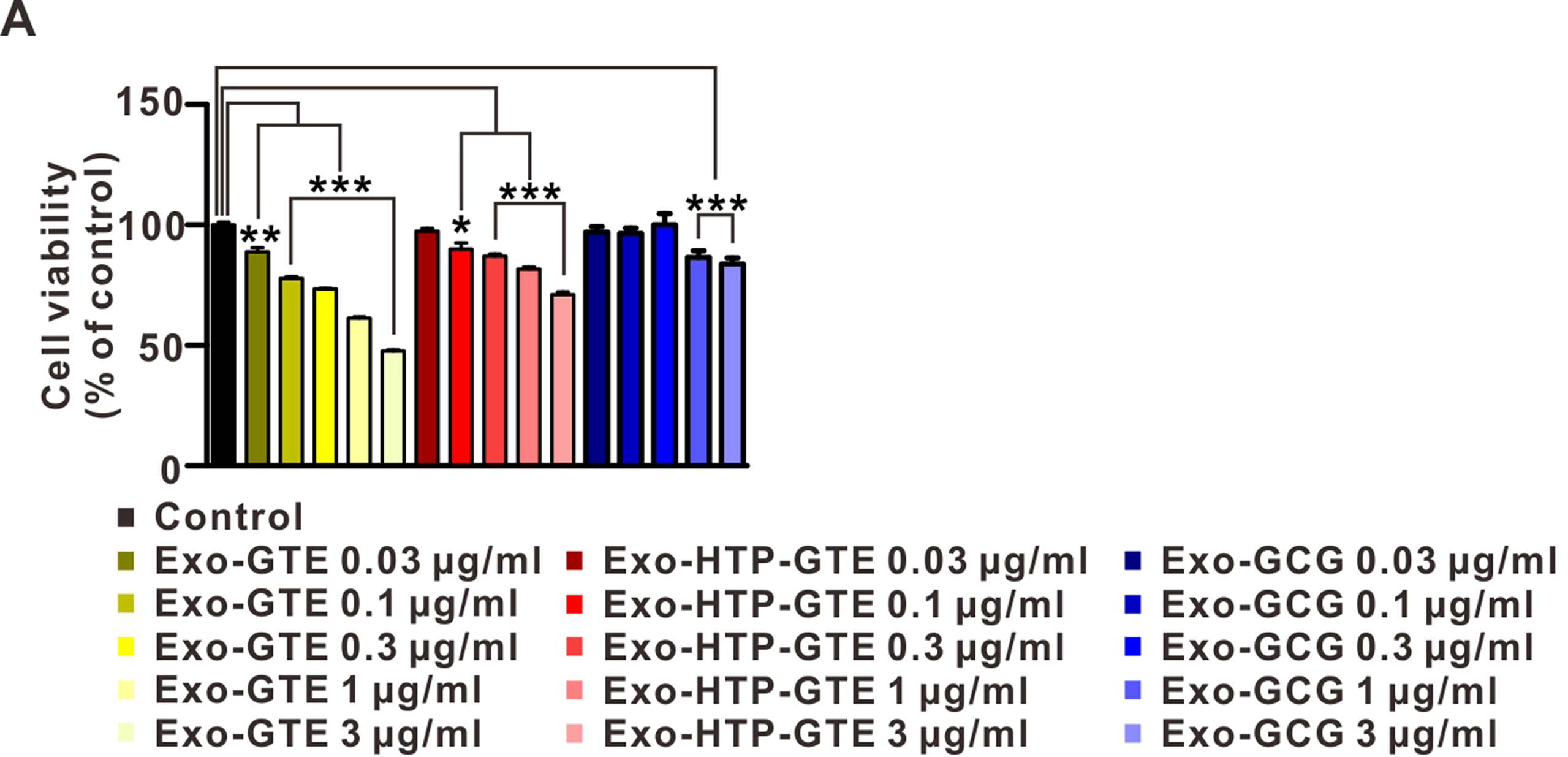

Supplement: figs3 [file mmcfigs3.jpg]

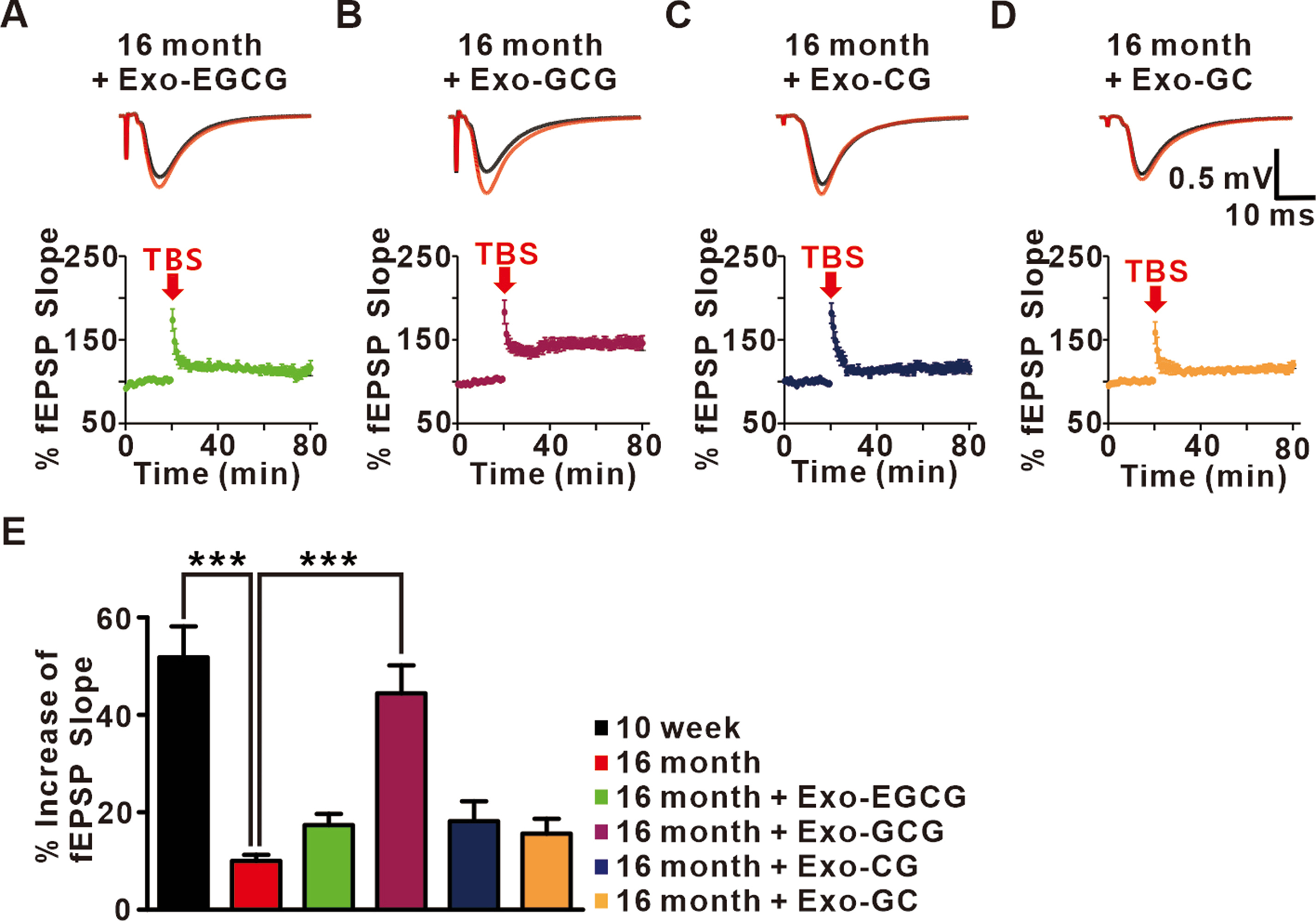

Supplement: figs4 [file mmcfigs4.jpg]

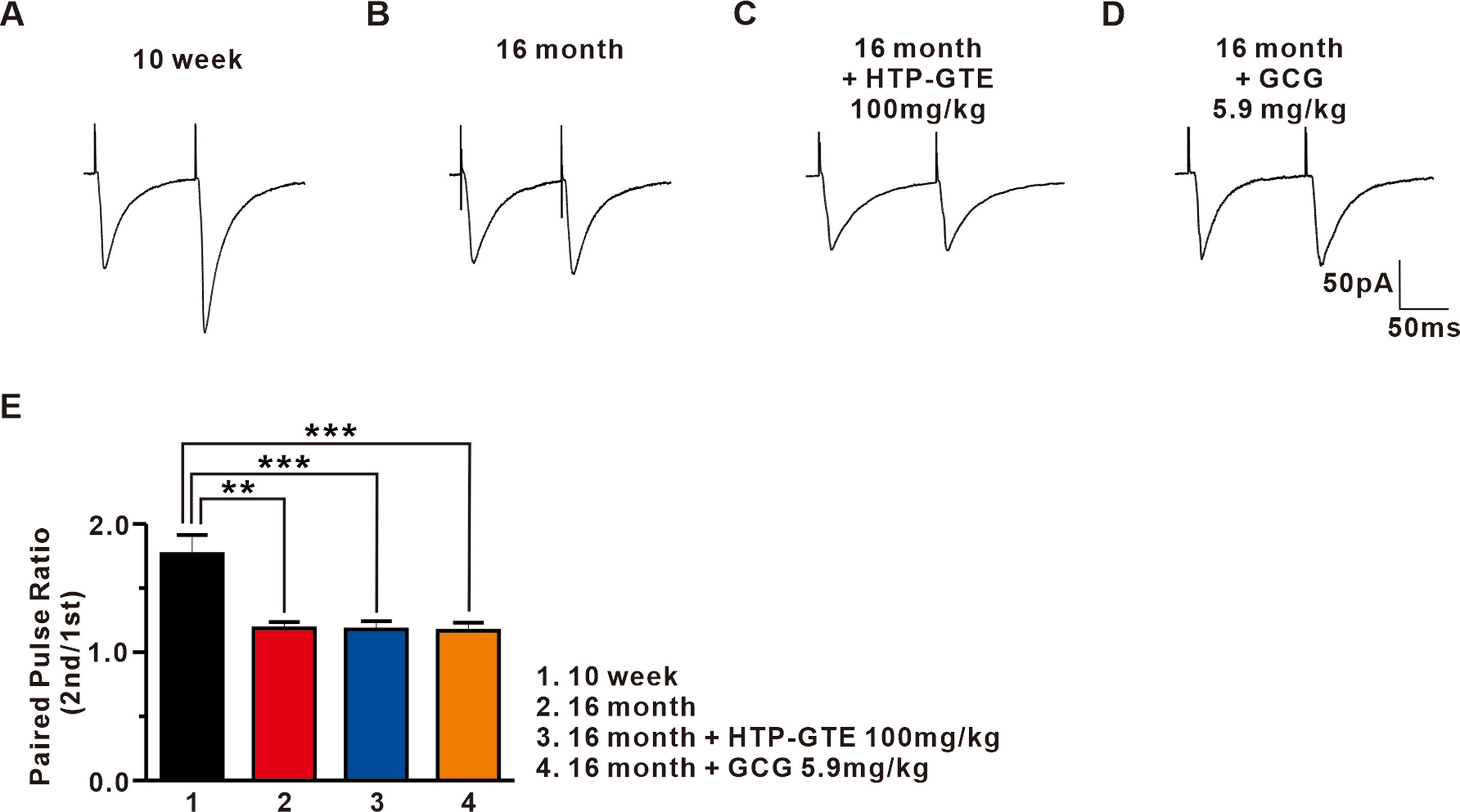

Supplement: figs5 [file mmcfigs5.jpg]

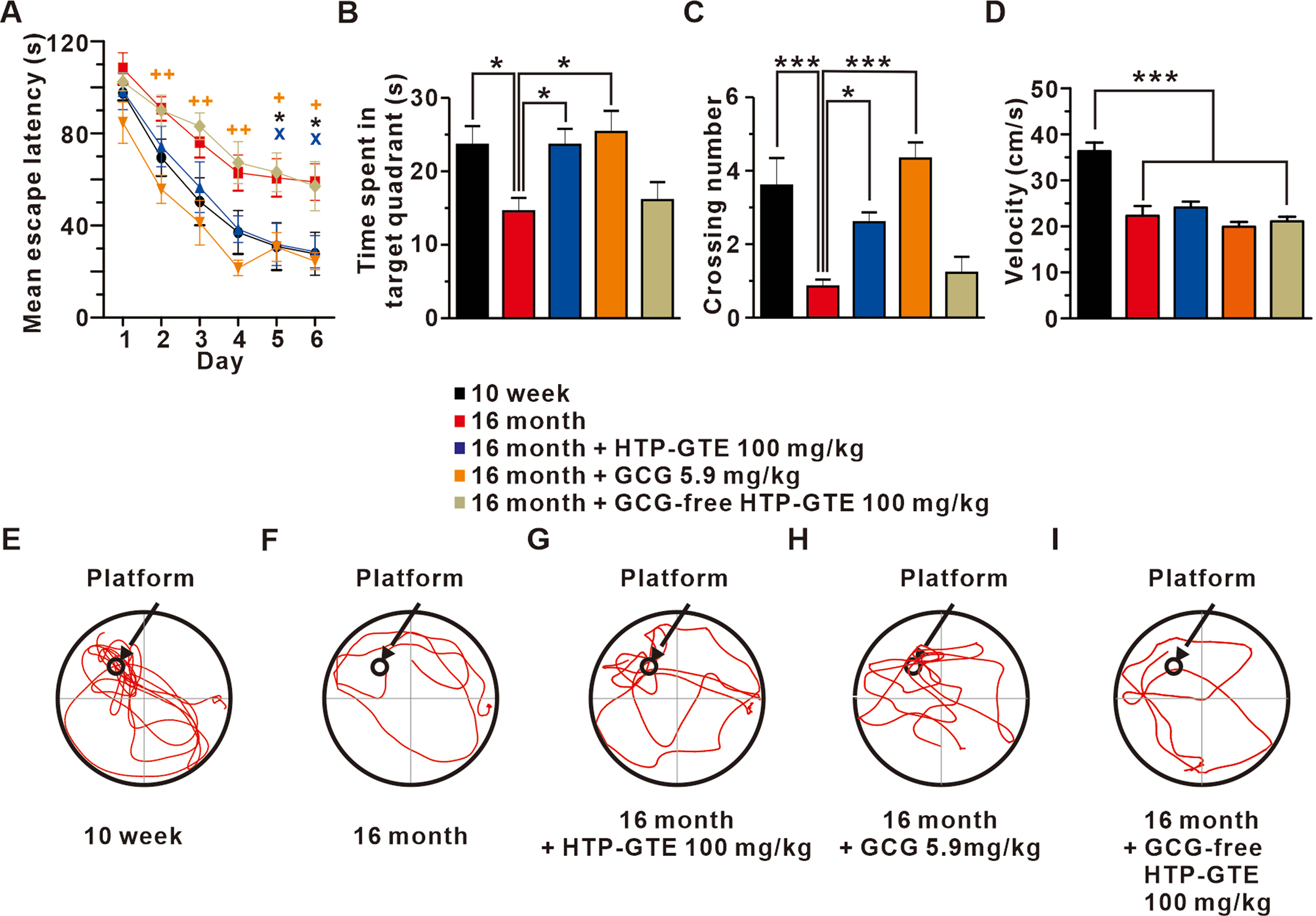

Supplement: figs6 [file mmcfigs6.jpg]

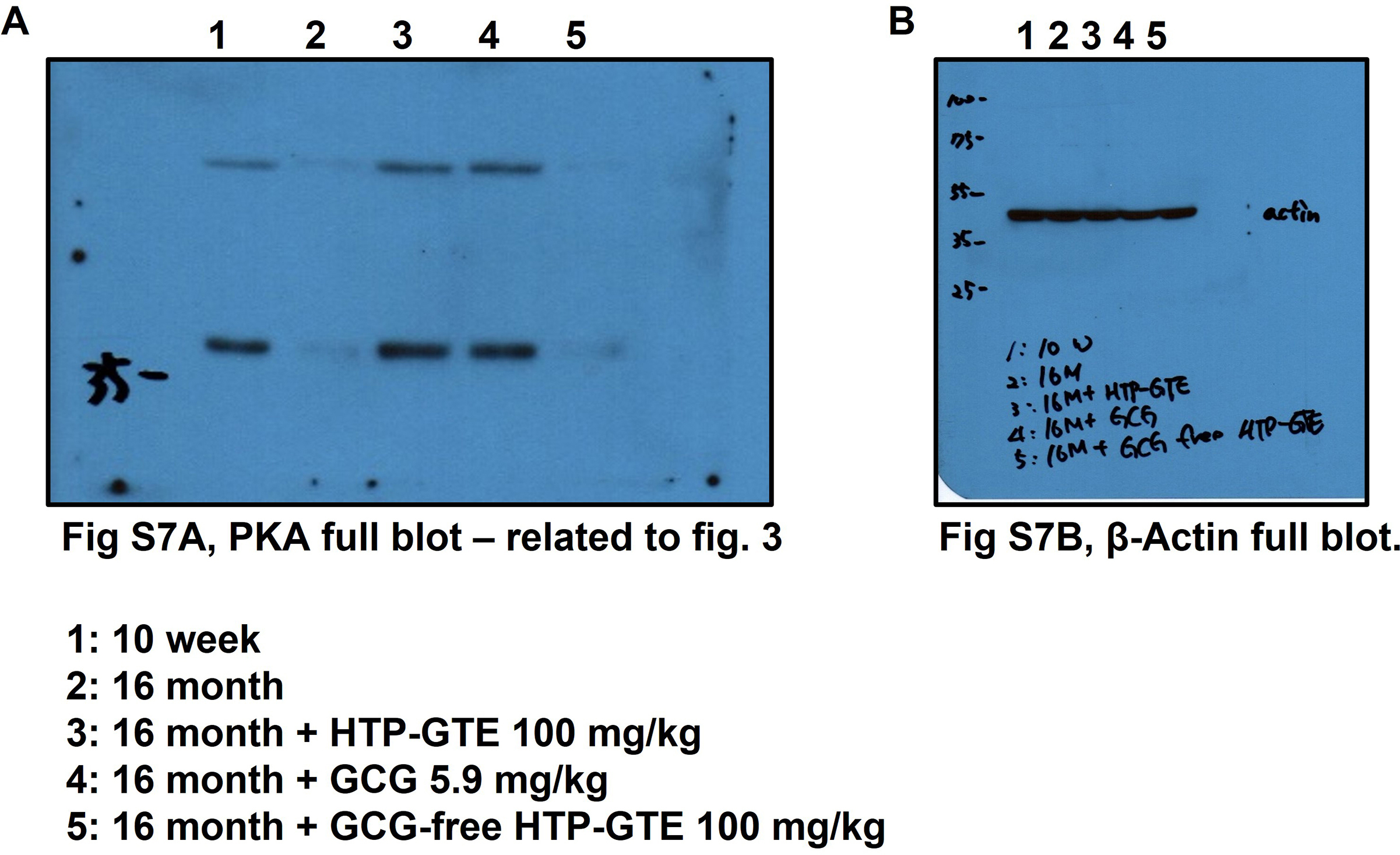

Supplement: figs7 [file mmcfigs7.jpg]

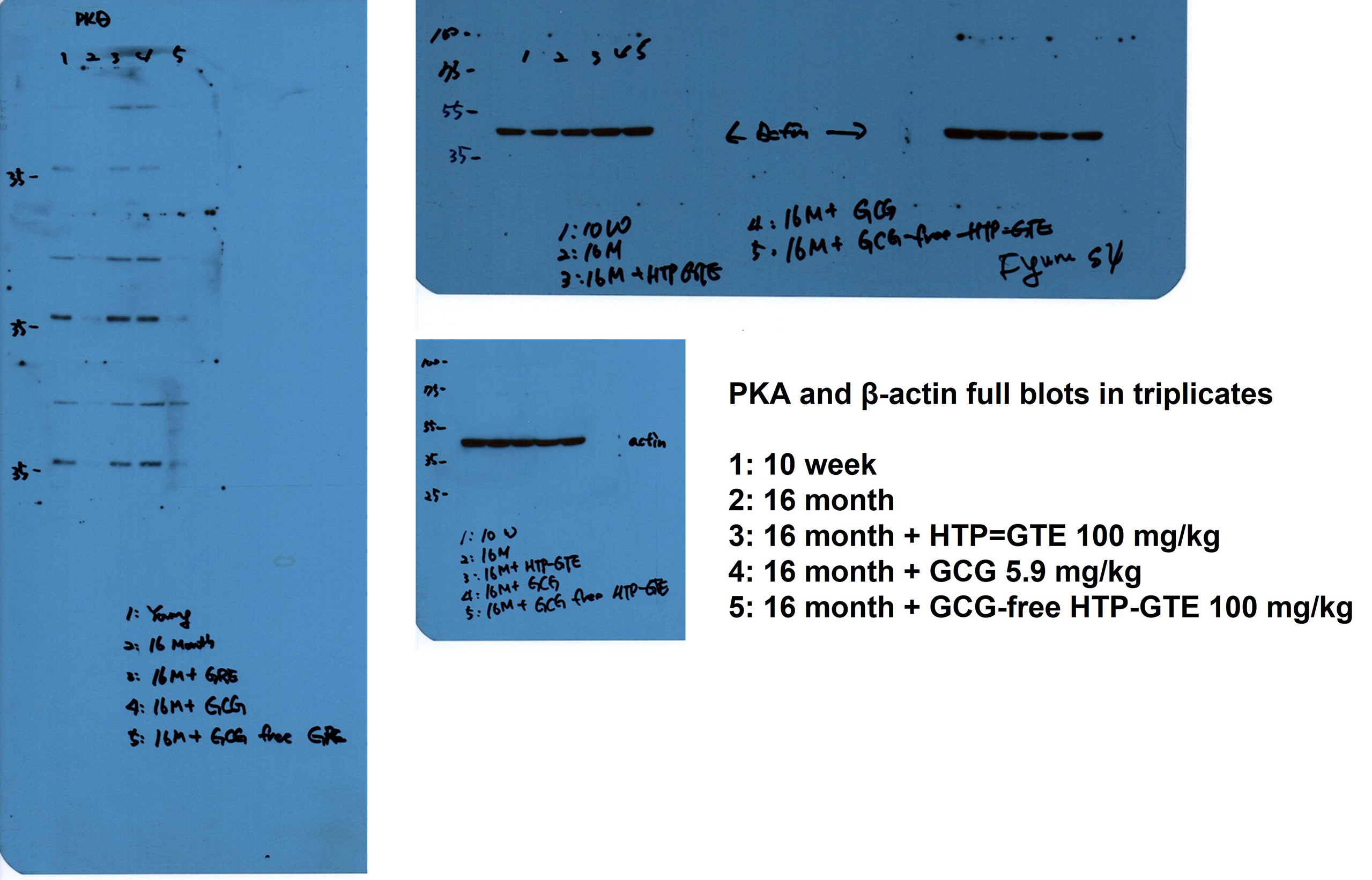

Supplement: figs8 [file mmcfigs8.jpg]
